# Which is Better in Fat Times and in Lean Times: the Macho Man vs. the Nice Guy? Priming Effects on Polish and Norwegian Students’ Mate Preferences

**DOI:** 10.1007/s12144-016-9530-3

**Published:** 2016-12-14

**Authors:** Natasza Kosakowska-Berezecka, Tomasz Besta

**Affiliations:** Institute of Psychology, University of Gdańsk, Bażyńskiego 4, 80-952 Gdańsk, Poland

**Keywords:** Social change, Social judgements of gender atypical behaviour, Backlash

## Abstract

Gender stereotypes serve as psychological tools that justify and maintain social inequality and reinforce the widely recognized status quo. Agency and anti-femininity are two widely prescribed qualities for men across cultures, leading them to refrain from engaging in household duties and parental roles (also referred to as communal roles). Several studies have documented backlash against men who engage in communal roles, but little attention has been given to the cultural and contextual cues influencing the perceptions of men who violate gender-norm prescriptions. Our study was conducted in two countries differing with regard to gender equality indices relating to extent to which men are allowed to manifest gender atypical behavior and influencing mate preferences of women. Polish (*N* = 106) and Norwegian (*N* = 77) female students were first presented with information which either a) threatened the stability of their country or b) highlighted the prosperity of their country. The participants were then asked to rate their romantic interest in the dating profiles of agentic (gender typical) and communal (gender atypical) men. Polish women who were provided with system-prosperity information found communal men to be more attractive than agentic men. This effect was not observed in the Norwegian sample; however, when provided with system-threat information, Norwegian students preferred agentic men over communal ones. Our results indicate that there exist certain contextual cues that might change perceptions of gender typical and gender atypical behavior.

## Culture and Gender Roles

Stereotypically, agency is associated with masculinity, and communality with femininity (Kite et al. 2008; Wood and Eagly 2012). The concept of masculinity encompasses a set of behaviors and traits that, in most cultures, entail agency and efficacy (Vandello and Bosson 2013). A man must be strong, concentrate on his career and achieve professional success, and if he fails to conform to social demands for agency, he risks backlash—social and economic penalties for gender atypical behavior (Kosakowska-Berezecka and Karasiewicz 2014; Kosakowska-Berezecka et al. 2016b; Rudman and Mescher 2013). According to biosocial construction theory (Wood and Eagly 2012), the division of labor between women and men is also maintained across societies through gender role beliefs that justify and normalize this division. If both sexes occupy roles in more equal proportions, then their sex traits are considered similar, and gender stereotypes are weaker (Glick and Fiske 2001; Wood and Eagly 2012). Lower gender stereotyping can also result in less risk of backlash (Carlsson et al. 2014; Kosakowska-Berezecka et al., under review) and higher attractiveness ratings for communal, non-agentic men (Eastwick et al. 2006).

Contemporary literature displays a clear focus on backlash against women who engage in atypical roles (e.g. Croft et al. 2015; Rudman and Mescher 2013), manifested in a preference for benevolent, communal women over gender atypical, agentic women (cf. Lau et al. 2008). However, relatively limited research has focused on the backlash against communal men (Croft et al. 2015; Kosakowska-Berezecka et al. 2016a; Rudman and Mescher 2013) and their attractiveness to women, especially from a cross-cultural perspective.

## Women’s Mate Preferences

Social context theories (e.g. Eagly 1987; Regan et al. 2000) stress that women seek partners with attributes consistent with gender-role stereotypes. However, recent research suggests that women might find the so-called nice guy more attractive than agentic men (Urbaniak and Kilmann 2006). Women often express an intention to date communal men that are kind, sensitive, and emotionally mature men rather than more traditional, tough, insensitive macho men. Research also indicates that kindness and niceness are among the most desired attributes in romantic partners (e.g. Buss and Angleitner 1989; Goodwin 1990).

However, the extent to which women perceive nice communal guys as attractive might depend on cultural and contextual factors. Social role theory predicts that traditional gender ideology is linked to sex-typing of mate preferences that are based on traditional gender stereotypes (Eagly et al. 2000). Further evidence for the contextual regulation of women’s mate preferences can be found in cross-cultural research which demonstrates that the more traditional gender ideology that participants manifest, the greater sex-typing of mate preferences they exhibit. Thus, the weaker traditional gender ideology is in a given country, the weaker stereotyping in mate preferences (Eastwick et al. 2006).

Perceptions of communal gender atypical communal men and agentic, macho men, thus, depend on the extent to which men are allowed to manifest gender atypical behaviour, which varies among cultures. How attractive this new, gender atypial behavior is to women actually sets the standards for what may be demanded from men.

## Threats to the System and Mate Preferences

According to system-justification theory, people encountering threats to their sociopolitical system are motivated to restore their belief in that system by engaging in psychological processes that strengthen its legitimacy (Jost et al. 2004). Endorsement of stereotypes that justify a given system are examples of such system-justifying psychological processes (e.g. Kay et al. 2007). Lau et al. (2008) argued that, when people experience a threat to the legitimacy of their sociopolitical system, their mate preferences might be affected by whether a potential romantic mate manifests traits and behavior embodying system-justifying stereotypes. Their results showed that men who experienced system threat had greater romantic interest in gender typical women who embodied stereotypical feminine ideals than men who did not experience system threat (Lau et al. 2008). As well, men who experienced system threat had greater romantic interest in gender typical women than gender atypical women (Lau et al. 2008). Therefore, in times of system threat, people might be more willing to engage in psychological processes that restore the desired legitimacy (Jost et al. 2004). Also, according to terror management theory (TMT) (Greenberg and Kosloff 2008) in order to maintain psychological security when faced with their personal mortality, individuals need to maintain faith in their sociopolitical systems and cultural values. TMT research shows that, when reminders of death (e.g. caused by threats to sociopolitical systems) heighten the psychological need to defend worldviews, gender-related prejudice and gender stereotyping increases.

In our study, we speculate that the cultural context can be related to mate selection and that priming certain aspects of individuals’ socio-political systems (as well as threatening the system) can lead to differences in the perceptions of attractiveness of agentic (gender typical) and communal (gender atypical) men. Stereotypes of men can serve as psychological tools that determine which characteristics of men and women are valued in a society (Cuddy et al. 2015; Jost and Kay 2005). Gender stereotypes are weaker in gender-egalitarian countries; thus, the attributes associated with the ideal man depend on what is encompassed within the concept of masculinity in a given country.

In our study, we connect the processes of system justification with mate preferences and suggest that the priming effects are different depending on the culture in which they are observed. We focus on romantic preferences for agentic (gender typical) or communal (gender atypical) men in two countries with different gender-equality levels (Norway and Poland), which in turn might relate to extent to which gender atypical behaviour in men is attractive to women. Norway is considered to be a model gender-egalitarian country (World Economic Forum 2015) and ranks 2nd in the world in the Global Gender Gap Report, with a score of 0.85 (Global Gender Gap Index (GGGI, World Economic Forum 2015). In contrast, Poland ranks 51st and has a score of 0.72 on the GGGI (World Economic Forum 2015).

As gender stereotyping is lower in gender egalitarian countries such as Norway we assume that women in Norway rate communal men as more attractive than agentic men and that Polish women prefer agentic men over communal men (hypothesis 1). Based on research on sociopolitical system threat we also hypothesize that, independent of culture, female participants whose system is threatened show greater romantic interest in men who embody stereotypical ideals of agentic, macho men than those who do not embody these ideals and are more soft and communal (hypothesis 2). We also predict that women exhibit greater interest in the softer and communal men when their system is secure and prosperous (hypothesis 3). We assume that differences in romantic interest in gender typical men and gender atypical men are dependent on perceived social-system conditions, and we expect a two-way interaction between perceptions of system threat and the type of descriptions of men.

We also explore the role of participants’ cultural background and examine whether culture influences perceptions of attractiveness. Based on previous research, we speculate that that the perceived attractiveness of agentic and communal men differs by culture (hypothesis 4). Therefore, in the system-threat condition, Norwegian women are expected to prefer agentic men over communal ones more strongly than Polish women. In the prosperity condition, we expect that Polish women prefer communal men over agentic ones less so than Norwegian women.

## The Present Study

### Method

#### Participants and Procedure

Female undergraduate students from Poland (*N* = 106; University of Gdansk) and Norway (77; University of Stavanger) voluntarily participated in the study. After a short introduction explaining the goals of the study (determining the relationship between social perceptions and the clarity of a text), participants read instructions which asked them to judge the clarity of a text, followed by an excerpt from a newspaper. The text differed depending on the experimental condition. The excerpt used in the system-threat condition included information about global threats (i.e. threats to the security of participants’ country). The second excerpt praised the results of the success and prosperity of the socio-economic system and stated that the economic and political climate of the given country was stable, positive, and prosperous (cf. Kay et al. 2005; Lau et al. 2008). In the third condition, participants read a neutral text related neither to threats to their country nor to prosperity of social system. After responding to items evaluating the clarity and writing style of the texts, participants then were asked to rate their romantic interest in four men with profiles including a self-description ostensibly taken from a dating website. Half of the profiles portrayed the men as agentic, and half as communal.

#### Romantic Interest

Participants used a 7-point Likert type scale (1 = completely do not agree; 7 = completely agree) to respond to 8 questions relating to their romantic interest (e.g. They reported the extent to which they found a man attractive; would like to go on a date with him, cf. Lau et al. 2008). We then calculated an index of romantic interest in agentic and communal men (Cronbach’s alpha of the evaluations of the four profiled men ranged from .91 to .96).

To examine the role of cultural and contextual clues in mate preferences, we introduced three between-subjects conditions: control (neutral) condition, system-threat condition, and system-prosperity condition. In addition, women rated their romantic interest in both agentic (gender typical) men and communal (gender atypical) men. Thus, the study had a 2 (country: Poland vs. Norway) by 3 (social context conditions: threat vs. prosperity vs. control) by 2 (communal vs. agentic men; within-subject variable) design.

## Results and Discussion

To examine our hypotheses, we conducted general linear model analyses with between-subjects factors 2 (culture) by 3 (experimental conditions) and 2 (within-subject measures of romantic interest in communal vs. agentic target men). The analyses showed no effect of culture - ther were no differences in the attractiveness of the target men in the control groups in either the Polish sample (agentic men: *M* = 2.92, *SD* = 1.06; communal men: *M* = 2.85, *SD* = 1.16; *t*(33) = .50, *p* = .62, *d* = .09) or the Norwegian sample (agentic men: *M* = 3.47, *SD* = 1.45; communal men: *M* = 3.25, *SD* = 1.45; *t* (21) = 1.17, *p* = .26, *d* = .25), thus, H1 was not supported. Moreover, we found a significant interaction effect between description of men (agentic vs. communal) and threat to the social system (threat vs. prosperity vs. control condition), with *F*(2177) = 3.57, *p* = .03, η^2^ = .04. When the Polish and Norwegian samples were both considered, H2 was not confirmed because differences in the perceived attractiveness of agentic and communal men did not reach significance (*M* = 3.65, *SD* = 1.24 vs. *M* = 3.44, *SD* = 1.17, respectively; paired-samples t-test *t*(58) = 1.05, *p* = .30, *d* = .14) (For *d* calculations, we corrected for dependence between means using Morris and DeShon’s (2002) Eq. 8). With regard to H3 our results showed that communal men were evaluated more positively than agentic ones in the prosperity condition but the difference was relatively small (*M* = 3.89, *SD* = 1.47 vs. *M* = 3.56, *SD* = 1.29, respectively; paired t-test *t*(67) = 1.96, *p* = .055, *d* = .24).

**Fig. 1:**
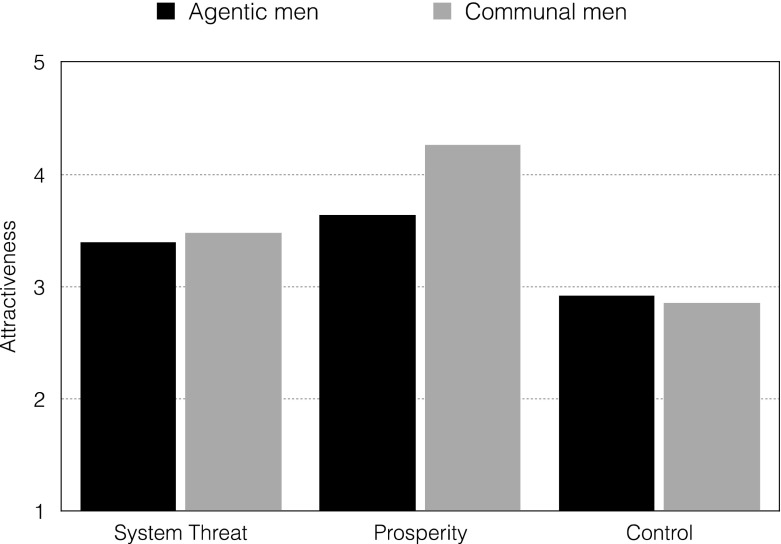
Mean ratings of romantic interest in agentic and communal men in the system-threat and system prosperity and control conditions – Polish sample

To test H4 and explore differences in romantic interest in agentic and communal men depending on experimental manipulation (system threat vs. system prosperity vs. control) in both countries, we conducted separate paired-samples t-tests in the Polish and Norwegian samples. The results showed that Polish participants showed significantly greater romantic interest in communal men (*M* = 4.26, *SD* = 1.31) than agentic men (*M* = 3.64, *SD* = 1.22) but only in the prosperity condition (*t* (36) = −2.58, *p* = .01, *d* = .42). This difference was not significant in the system-threat condition (agentic men: *M* = 3.39, *SD* = 1.14; communal men: *M* = 3.48, *SD* = 1.24; *t* (34) = .40, *p* = .70, *d* = .06) (see Fig. 1).

**Fig. 2:**
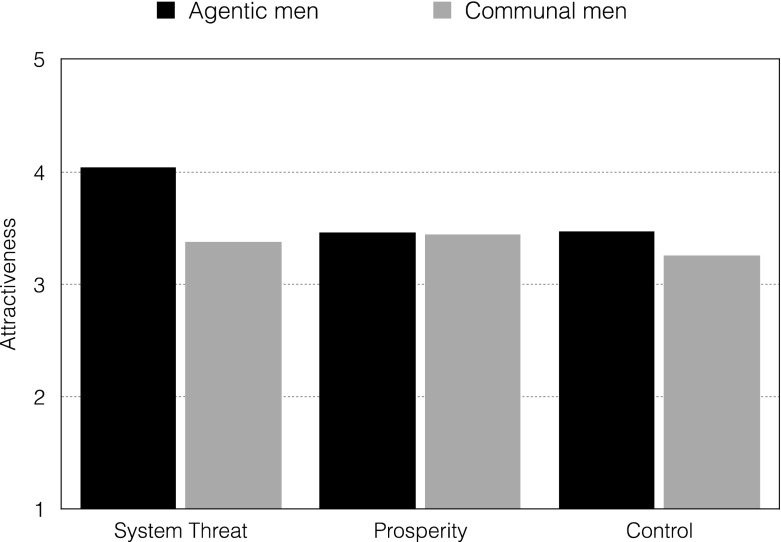
Mean ratings of romantic interest in agentic and communal men in the system-threat and system prosperity and control conditions – Norwegian sample

In the case of the Norwegian sample, this difference was significant only in the system-threat condition (*t* (23) = 2.05, *p* = .05, *d* = .42), with participants showing greater romantic interest in agentic men than communal men (*M* = 4.04, SD = 1.29; *M* = 3.38, *SD* = 1.09, respectively). This difference was not significant in the prosperity condition (agentic men: *M* = 3.46, *SD* = 1.38; communal men: *M* = 3.44, *SD* = 1.56; (30) = .06, *p* = .95, *d* = .02) (Fig. 2).

The results show that, in both Polish and Norwegian control groups, no differences in romantic interest in agentic and communal men were observed. The two types of men were perceived as equally attractive romantic partners by Polish and Norwegian women; hence, H1 was not confirmed. Only when priming was induced did differences in romantic interest in agentic and communal men emerge. However, H2 found confirmation only in the Norwegian sample, and H3 was confirmed in the Polish sample. An effect of system-threat was visible only in the Norwegian sample as priming threat to the socioeconomic system led women to show greater romantic interest in agentic, gender typical men than soft, communal men. Priming prosperity had no effect on romantic interest in agentic and communal men in the Norwegian sample, likely because this condition was the default system in which Norwegian participants lived - Norway has one of the highest scores on the Organisation for Economic Co-operation and Development OECD (2014).

In the case of the Polish sample, the group that read the text describing the prosperity of the Polish country showed greater romantic interest in communal men, while the system-threat condition had no effect on communal-vs.-agentic-men ratings. As prosperity could be the default system for Norway, then system-threat might be the default position of the Polish system. Polish citizens exhibit lower levels of social and political trust, feel less secure economically than Norwegians (ESS Round 7: European Social Survey Round 7 Data 2014), and have lower scores on the OECD (2014). Therefore, threats to the system do not have effects because they might be experienced as the regular state of Poland. System-threat, however, is not default state for Norway, which is considered to have high national prosperity and welfare.

In our study we have not controlled for participants sexual orientation and current relationship status and these variables could be controlled for in follow-up studies.

Our results add to the research stream on the contextual and cultural cues that influence both gender stereotyping and women’s mate preferences. Romantic interest in gender typical men and gender atypical men depends on participants’ perceived sociopolitical system conditions and their cultural background. Therefore, a cross-cultural perspective is important to produce a more nuanced theory of the psychological processes underlying gender stereotyping and mate preferences.
